# The Dual Task Ball Balancing Test and Its Association With Cognitive Function: Algorithm Development and Validation

**DOI:** 10.2196/49794

**Published:** 2024-08-19

**Authors:** Barry Greene, Sean Tobyne, Ali Jannati, Killian McManus, Joyce Gomes Osman, Russell Banks, Ranjit Kher, John Showalter, David Bates, Alvaro Pascual-Leone

**Affiliations:** 1 Linus Health Boston, MA United States; 2 Linus Health Europe Dublin Ireland; 3 Hinda and Arthur Marcus Institute for Aging Research Deanna and Sidney Wolk Center for Memory Health Hebrew SeniorLife Boston, MA United States

**Keywords:** cognitive function, dual task, inertial sensors, mHealth, tablet, MCI, Alzheimer, dementia, motor, older adults, cognitive impairment, balance

## Abstract

**Background:**

Dual task paradigms are thought to offer a quantitative means to assess cognitive reserve and the brain’s capacity to allocate resources in the face of competing cognitive demands. The most common dual task paradigms examine the interplay between gait or balance control and cognitive function. However, gait and balance tasks can be physically challenging for older adults and may pose a risk of falls.

**Objective:**

We introduce a novel, digital dual-task assessment that combines a motor-control task (the “ball balancing” test), which challenges an individual to maintain a virtual ball within a designated zone, with a concurrent cognitive task (the backward digit span task [BDST]).

**Methods:**

The task was administered on a touchscreen tablet, performance was measured using the inertial sensors embedded in the tablet, conducted under both single- and dual-task conditions. The clinical use of the task was evaluated on a sample of 375 older adult participants (n=210 female; aged 73.0, SD 6.5 years).

**Results:**

All older adults, including those with mild cognitive impairment (MCI) and Alzheimer disease–related dementia (ADRD), and those with poor balance and gait problems due to diabetes, osteoarthritis, peripheral neuropathy, and other causes, were able to complete the task comfortably and safely while seated. As expected, task performance significantly decreased under dual task conditions compared to single task conditions. We show that performance was significantly associated with cognitive impairment; significant differences were found among healthy participants, those with MCI, and those with ADRD. Task results were significantly associated with functional impairment, independent of diagnosis, degree of cognitive impairment (as indicated by the Mini Mental State Examination [MMSE] score), and age. Finally, we found that cognitive status could be classified with >70% accuracy using a range of classifier models trained on 3 different cognitive function outcome variables (consensus clinical judgment, Rey Auditory Verbal Learning Test [RAVLT], and MMSE).

**Conclusions:**

Our results suggest that the dual task ball balancing test could be used as a digital cognitive assessment of cognitive reserve. The portability, simplicity, and intuitiveness of the task suggest that it may be suitable for unsupervised home assessment of cognitive function.

## Introduction

Recent research has suggested that up to 40% of dementia cases [1] can be delayed or prevented through early identification of impairment and adherence to recommended lifestyle modifications [2]. Furthermore, recent developments in pharmaceutical intervention suggest that the progression of Alzheimer dementia can be delayed through amyloid plaque removal [3].

An individual's cognitive and behavioral performance is a combination of brain activity and cognitive reserve. Cognitive reserve can be conceptualized as a property of the brain that allows for better than expected performance, given the degree of life-course related brain changes and brain injury or disease [4]. Cognitive reserve can be influenced by multiple genetic and environmental factors, operating at various points or continuously across the lifespan. In the presence of disease, for example, a neurodegenerative disease such as Alzheimer disease, cognitive reserve is engaged to sustain function for as long as possible and minimize symptoms and disability. Thus, individuals with more cognitive reserve manifest symptoms or disability later than those with lower cognitive reserve; symptoms are less prominent or severe than might be expected for a given amount of pathology. Low cognitive reserve makes individuals with underlying brain pathology prone to episodes of confusion, delirium, and other acute decompensations when exposed to a stressor or insult, for example, elective surgery, infection, sleep, and deprivation. Individuals with mild cognitive impairment (MCI) and higher cognitive reserve can delay the development of dementia. Thus, assessment of cognitive reserve is important to predict an individual’s functional state and prognosis. In addition, cognitive reserve can be a powerful therapeutic target, as increasing cognitive reserve might reduce disability.

The brain’s resource allocation capacity has been studied extensively and is thought to provide insight into cognitive reserve and depend on prefrontal function. However, the nature and causality of this relationship is not as well understood. Dual task paradigms have long been thought to unlock deficits in the allocation of prefrontal resources [5]. Recent studies [6-8] have examined the impact of a cognitive task (eg, backward counting) on a participant’s gait or balance, and thus, are dependent on peripheral nerve and musculoskeletal factors often affected in older adults. Furthermore, gait and balance analysis may not be suitable or safe for use with older adults or those with comorbidities such as osteoarthritis, neuropathies, etc. A validated tool that can support objective characterization and quantitative evaluation of cognitive reserve safely and reliably in older adults, as well as early identification of cognitive decline in nonclinical settings, could be of clinical benefit in more accurately identifying those patients who would benefit most from early and targeted intervention.

We introduce a novel test of motor control, coordination, and attention—the “ball balancing” test, in which an individual is asked to maintain the position of a virtual ball in the center of a circular target area. Task performance is measured by examining the position of a virtual ball on the screen of a touchscreen tablet, estimated using the inertial sensors embedded in the tablet. The test can be easily adapted to a dual task condition, for example, by asking the individual to balance the ball while simultaneously doing a different, attention demanding task. The test can be completed comfortably and safely in a sitting position. In an initial version of a dual task paradigm, an individual’s ball balancing test performance was assessed while simultaneously conducting a backward digit span test (BDST).

We aimed to examine the use of the ball balancing test under single and dual conditions [as quantified using the inertial measurement unit (IMU) sensors embedded in the target device] in assessment of cognitive reserve and identification of cognitive impairment. While this task (and other dual task paradigms) is not primarily aimed at serving as a means to classify cognitive function, one may predict there should be a loss of cognitive reserve between MCI and dementia, given that cognitive reserve would be “used up” to sustain cognitive function and ultimately be no longer sufficient to prevent progression of deficit, impact on activities of daily living (ADL), and thus transition from MCI to dementia. We report the performance of the task in classifying cognitive status according to 3 different outcome measures (consensus clinical judgment, Rey Auditory Verbal Learning, and Mini Mental State Examination). Given that the outcome measures are imperfectly mutually correlated, it can be assumed that they may contain complementary information pertinent to assessment of cognitive function, which can be leveraged to examine cognitive reserve deficits.

## Methods

### Ball Balancing Task

Participants were seated in a comfortable and supportive chair and asked to hold a touchscreen tablet device (iPad Pro, Apple) parallel to the ground and tilt the screen as needed to keep a virtual ball within a target area—the ball was not perturbed during the test unless the tablet was moved by the participant.

Participants were asked to balance a virtual ball on a touchscreen tablet screen, the subsequent movement is measured by the IMU sensors embedded in the tablet and used to calculate the position of the virtual ball on the tablet screen. The ball balancing test was completed under both single task (ball balancing alone) as well as under dual task conditions, with participants completing a single trial of each. The dual task involved asking the participant to complete the ball balancing test while simultaneously completing a BDST. In the BDST, the participant is played an audio sequence of 4 digits and is prompted to repeat them in reverse order. The single task was 20 seconds in duration while the dual task was 45 seconds in duration.

**Figure 1:**
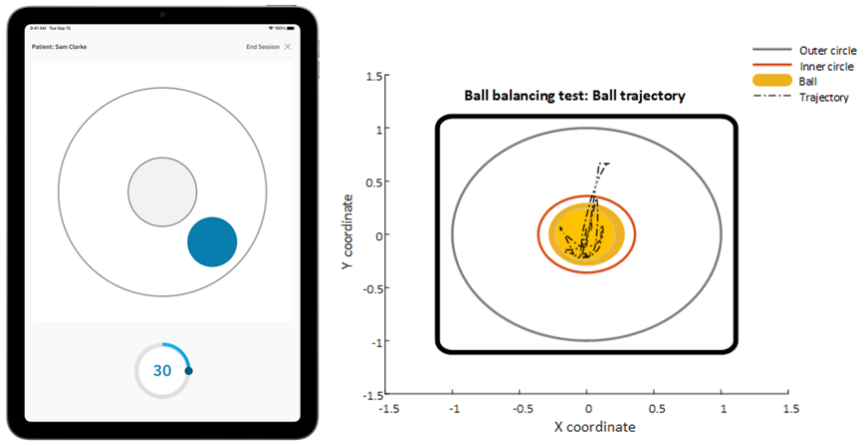
Linus Ball balancing task iOS app running on an iPad Pro (left panel). Trajectory of virtual ball position on screen over the course of a dual task ball balancing test, as calculated from tablet inertial measurement unit data (right Panel).

A custom iOS application (Swift, iOS) was developed to conduct the ball balancing test, supporting single and dual tasks. IMU and ball position data were stored within the application in JSON format and exported for offline analysis. All analyses were conducted using MATLAB (R2017b, MathWorks; Figure 1).

The following two distinct approaches were used to analyze the data, that is, by examining: (1) the ball position on the screen (estimated from IMU data); and (2) the IMU data from the movement of the tablet during the test.

### Signal Processing: Ball Position Metrics

The position of the ball on the screen was calculated using the inertial sensor data as input to a kinematic model, which derives the ball placement on the screen using Newtonian mechanics and allowed plotting of ball displacement on the screen. The following parameters were calculated from the virtual ball displacement (values in parenthesis indicate variants of the calculated feature) (Textbox 1).

For all ball position metrics, the displacement is normalized to the range [9], where the outer edge is the radius of the outer circle, while the radius of the inner circle is calculated based on the ratio of the inner circle radius to the outer circle. The percentage of time spent within the inner circle is calculated as the proportion of time where the resultant displacement is less than the radius of the inner circle less the radius of the ball. The radial symmetry is calculated as the sum of the first difference values of the resultant displacement from the center of the circle. It is intended to measure quadrant placement of the ball within the outer circle. To examine learning effects and changes in performance over the course of each test, the percentage of time within the inner circle is calculated for each 5-s epoch within the test. The mean, standard deviation, and first difference were then calculated across all epochs per test to provide a measure of intratest performance. A number of standard center of pressure measures [10,11] were also calculated based on time and frequency domain analysis of the ball displacement. Each ball position metric was calculated for each participant under single task (ST) and dual task (DT) conditions; the dual task cost was calculated as the percentage difference between the parameter value under DT conditions and the parameter value under ST conditions and can be expressed mathematically as –100*(DT-ST)/ST [12].

A “perfect score” was achieved when the ball was found to lie within the inner circle for 100% of the test. As it was possible to achieve a perfect score by placing the tablet flat on a table, we examined if perfect score tests had any effect on the overall results to rule out the possibility that certain participants were engaging less with the task but achieving a perfect score.

**Features calculated from the virtual ball displacement data for each ball balancing test.**

- Percentage of test time spent within inner circle
- Radial symmetry
- Percentage of time spent in the inner circle per 5-second epoch (mean, SD, and first difference)
- Median frequency of ball displacement (mean, X, and Y)
- 95% spectral edge frequency of ball displacement (mean, X, and Y)
- Sway area of ball displacement
- Mean sway frequency (mean, X, and Y)
- Mean sway distance (mean, X, and Y)
- Resultant sway distance (mean, X, and Y)
- Sway length of ball displacement (mean, X, and Y))
- Sway velocity (mean, X, and Y)

### Inertial Sensor Parameters

**Figure 2:**
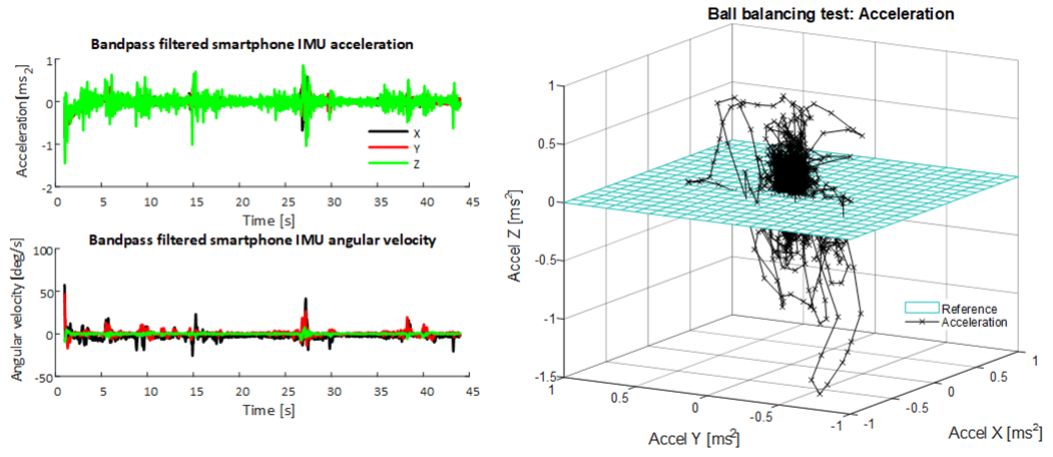
Inertial measurement unit (IMU) signal obtained from an iPad during a dual task ball balancing test (left panel). 3D acceleration signal about the tablet plane during a ball balancing test (right panel).

Inertial sensor data from the tablet device under both ST and DT conditions were processed using an adapted version of a previously reported algorithm [13,14]; this approach treats the IMU data as arising from motion about a rigid plane. Figure 2 below shows the IMU (triaxial accelerometer and triaxial gyroscope) data for a dual task ball balancing test.

For each test, 1 second of data was excluded from the start and end of each recording to remove artifacts due to tablet positioning. Any recordings less than 10 seconds were discarded. IMU data were resampled to 100 Hz as iPad IMU data can be unevenly sampled, leading to distortion in frequency domain signal features [15]. Signals were bandpass filtered using a fourth order Butterworth IIR filter, in the range 0.1-40 Hz and calibrated using a standard method [16].

The following parameters were calculated from the IMU data for each ball balancing test (Textbox 2).

For each calculated parameter, the dual task cost was calculated as the percentage difference between the parameter value under dual task conditions and the parameter value under single task conditions. Figure 2 provides a 3D representation of the ball balancing test signal relative to the rigid plane.

**Features calculated from inertial sensor data for each ball balancing test.**

- Root mean square (RMS) acceleration (m/s^2^)
- RMS acceleration—x-axis (m/s^2^)
- RMS acceleration—y-axis (m/s^2^)
- RMS acceleration—z-axis (m/s^2^)
- RMS angular velocity (°/s)
- Median frequency acceleration (Hz)
- RMS angular velocity—x-axis (°/s)
- RMS angular velocity-y-axis (°/s)
- Spectral edge frequency acceleration (Hz)
- Spectral entropy acceleration
- Median frequency angular velocity (Hz)
- Spectral edge frequency angular velocity (Hz)
- Spectral entropy angular velocity
- Sway path length of acceleration—x-axis (m/s^2^)
- Sway path length of acceleration—z-axis (m/s^2^)
- Sway area of the acceleration
- Sway jerk of the acceleration
- Area of 95% confidence ellipse of acceleration

### Statistical Analysis

To examine the association between the calculated ball balancing test parameters and cognitive function, we considered the 3 available neurocognitive measures (cohort status, Mini Mental State Examination [MMSE], and Rey Auditory Verbal Learning Test [RAVLT]), treated as either continuous variables or binary labels (eg, impaired or not impaired). Cohort status was treated as a 3-category label (healthy, MCI, and Alzheimer disease–related dementia [ADRD]). Similarly, the differences between the healthy and impaired subgroups (MCI and ADRD) were also examined using a Wilcoxon rank sum test. A Wilcoxon signed rank test was used to test for significant differences across task conditions.

The MMSE (total score) and RAVLT (long recall delay score) data were dichotomized into cognitively impaired and cognitively intact with values below a threshold of 28 for the MMSE [17] and age group thresholds for the RAVLT [18] used to identify impaired cognition.

Spearman rank correlation was used to examine the relationship between each feature with the MMSE and RAVLT, while the Wilcoxon rank sum test was used to test for differences between impaired and nonimpaired groups for each feature. A confusion matrix was calculated for each set of binary labels (impaired/nonimpaired) to see how well cohort status, MMSE-, and RAVLT-based categorization agree with each other.

To examine the association of each variable with cognitive function and allow for the effect of age, a linear mixed effects model analysis was conducted with age as a within-subjects’ factor and cohort status as a categorical response variable. ANOVA was then used to examine the effect of each factor on cohort status, while controlling for age. This analysis was repeated for each variant with binary cohort status as well as impaired and nonimpaired labels obtained from MMSE and RAVLT.

In addition, we aimed to examine if any of the calculated ball balancing parameters were associated with functional impairment, independent of cognitive impairment. We conducted a one-way ANOVA for each ball balancing parameter with functional impairment (as measured by the Functional Activity Questionnaire [FAQ], with a threshold greater than or equal to 6 denoting functional impairment), controlling for MMSE and age. This analysis was then repeated when controlling for RAVLT and age.

To determine how well ball balancing parameters (features) could classify “unseen” participants according to binary cognitive status (cognitively impaired or cognitively intact), we used a logistic regression classifier model with a sequential forward feature selection procedure [19] validated using 10-fold cross-validation. Interaction terms were included in the candidate feature set and separate models were produced for each condition and feature set (ST, DT, dual task cost, all features as well as age only).

### Data

A sample of 375 older adults (n=210 female; aged 73.0, SD 6.5 years). Completed a battery of cognitive and motor function tests as part of wider study on brain health. The Bio-Hermes research study is managed by the Global Alzheimer Platform (GAP) and seeks new solutions to monitor and maintain brain health. Each participant received a clinical examination, which included the MMSE [20], the RAVLT [21] and “cohort status,” which classified participants into 3 clinical categories (healthy, MCI, and ADRD), as determined by a panel of qualified clinicians. For RAVLT, 2 summary scores were examined: the RAVLT total score and the RAVLT long recall delay score. In addition, each participant completed an FAQ [9] to examine functional status including ADL.

### Ethical Considerations

The Bio-Hermes research study is managed by the GAP. The study was performed in accordance with the Declaration of Helsinki and its later amendments. The study procedures were explained to participants verbally and through written informed consent that was approved by the local IRB of each site participating in the GAP consortium (see the Bio-Hermes study website [22] for a list of study sites). If, in the opinion of the site principal investigator, the participant did not have the capacity to sign the informed consent form, a legally authorized representative was used to grant consent on behalf of the participant. Ethical approval was granted by each institution participating in the GAP consortium (reference number: Pro00046018). Inclusion criteria for the study were adults 60-85 years of age, fluent in the language of the tests used and the test site, and with an MMSE score of 20-30 at Screening. Exclusion criteria were extensive and based on underlying conditions. All data collected as part of this study were deidentified to confidentiality protection. Participants in the study were compensated in order to cover any time or expense they incurred as a result of completing the study.

## Results

**Figure 3:**
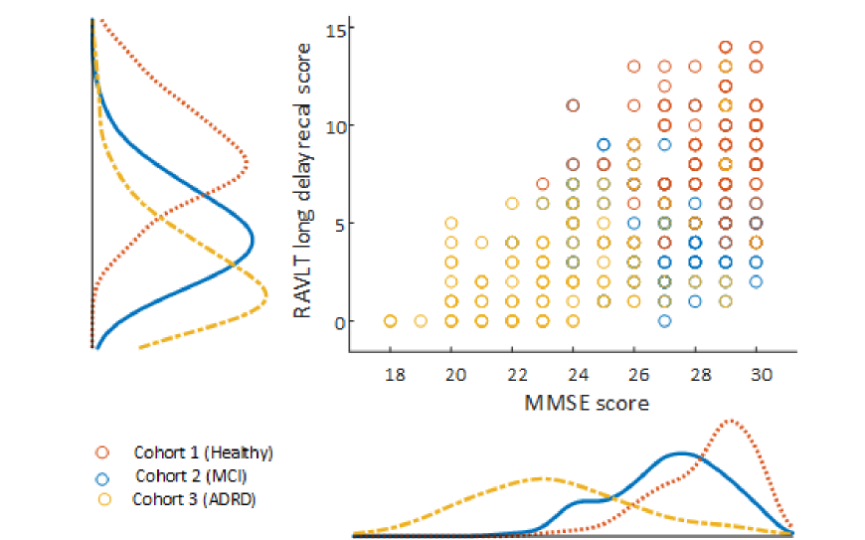
Relationship between the Mini Mental State Examination (MMSE) and Rey Auditory Verbal Learning Test (RAVLT) long recall delay score for Bio-Hermes population sample. Pearson correlation coefficient of 0.60 was observed between the 2 outcome measures. ADRD: Alzheimer disease–related dementia; MCI: mild cognitive impairment.

Age was significantly different (*P*<.001) across cohort status groups. The mean total MMSE scores for the sample was 26.3 (SD 3.0), mean total adjusted RAVLT score was 38.8 (SD 14.3), while mean RAVLT long delay score was 5.4 (3.5). According to cohort status, 132 participants were deemed cognitively normal, 116 were considered to have MCI and 126 had probable AD (ADRD), 1 participant did not have a valid cohort status label. Combining the MCI and ADRD classes to produce 2 classes (Impaired and Intact) produced 242 participants with cognitive impairment and 132 deemed intact. Using MMSE and FAQ cut-offs of 28 and 6, respectively, along with RAVLT age group thresholds [18] to categorize participants as impaired or unimpaired, allowed a comparison of these labels against binary cohort status. MMSE agreed with cohort status with 73.8% (277/375) accuracy, RAVLT total score agreed with cohort status 45.7% (171/375), while RAVLT long recall delay score agreed with 84.8% (318/375) accuracy. Pearson's correlation coefficient between MMSE and RAVLT total score was 0.43, while correlation coefficient between MMSE and RAVLT long delay recall score was 0.60 (see Figure 3).

### Ball Balancing Task Performance

**Figure 4:**
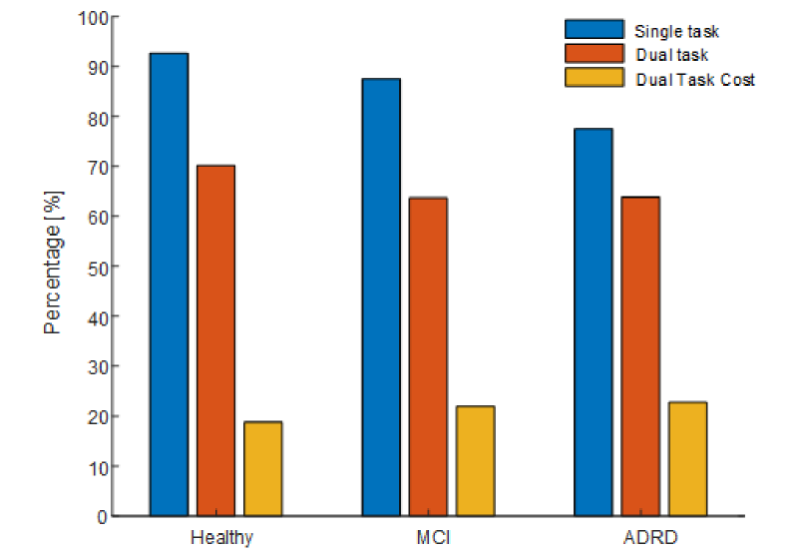
Ball balancing task performance measures per cohort status group and task condition.

**Figure 5:**
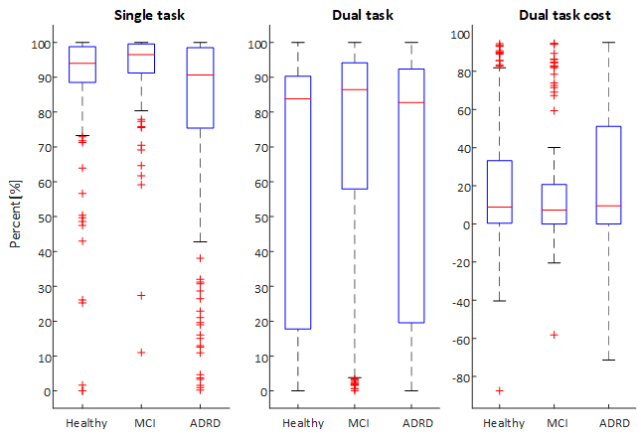
Box plot of ball balancing test performance (percentage of time within inner circle) under single (left panel) and dual (center panel) task conditions as well as dual task cost (right panel) for each cohort group (Healthy, mild cognitive impairment [MCI], and Alzheimer disease–related dementia [ADRD]).

All participants were able to complete the task under ST and DT conditions. The main metric of task performance was the percentage of time the virtual ball spent within the inner circle (“percentage time in circle”). Mean percentage time spent in the inner circle was 86.0% (SD 23.0%) and 66.1% (SD 35.8%) under ST and DT conditions, respectively, while the mean DT cost was 21.0% (SD 34.1%). Task performance was significantly different (*P*<.05) across cognitive status groups and between ST and DT conditions (Table 1). As expected, participants achieved lower performance under DT conditions with a higher mean percentage time within the inner circle and a lower proportion of “perfect score” tests (see Figures 4 and 5). Removing tests with “perfect” task performance (percentage time is circle equal to 100%) did not change this finding. Performance in the task declined with increased cognitive impairment, with best mean performance observed in the healthy group for both ST and DT and worst task performance in the ADRD group.

Task performance was statistically significantly different (*P*<.05) across group and between conditions.

**Table 1:** Ball balancing task performance: percentage (%) of time spent within the circle for each of the cohort groups (all participants, healthy, mild cognitive impairment [MCI], and Alzheimer disease–related dementia [ADRD]).

Group	Single task performance (%), mean (SD)	Dual task performance (%), mean (SD)	Dual task cost (%), mean (SD)
All	86.0 (23.0)	66.1 (35.8)	21.0 (34.1)
Healthy	92.6 (12.2)	70.2 (34.0)	18.8 (29.7)
MCI	87.5 (20.4)	63.7 (37.0)	21.9 (35.4)
ADRD	77.5 (30.0)	63.9 (35.9)	22.7 (37.1)

### Exploratory Results for Cohort Status

Age is significantly different between impaired and nonimpaired groups. When controlling for age using ANOVA, a large number of calculated parameters below were significantly (*P*<.05) different on the basis of 3 category cohort status.

**Figure 6:**
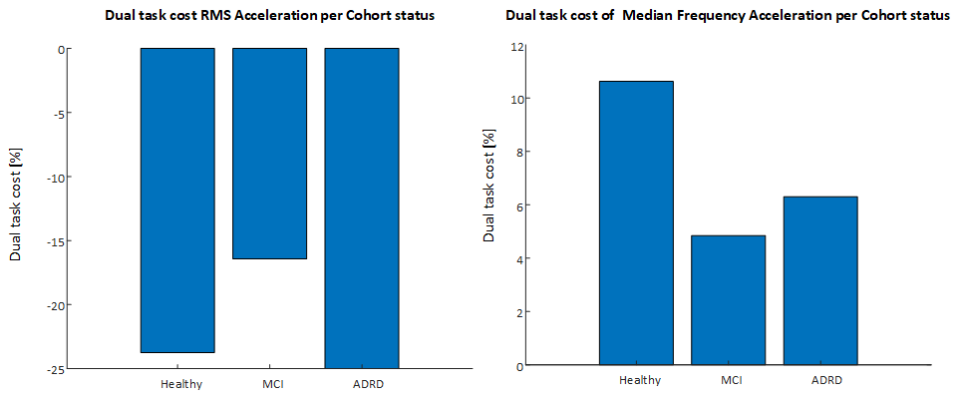
Dual task cost of RMS acceleration and median frequency acceleration per cohort status. ADRD: Alzheimer disease–related dementia; MCI: mild cognitive impairment; RMS: root mean square.

Similarly, when using ANOVA with a binary cohort label and correcting for age, a large number of parameters were significantly (*P*<.05) different on the basis of binary cognitive status. Figure 6 below details 2 IMU parameters where there were significant differences across groups when corrected for age.

### Classification Using Cohort Status

A linear logistic regression classifier model based on ball balancing measures from the DT condition (including age and gender) compared against a model obtained from age only found that ball balancing parameters could classify cognitive status with 70.5% accuracy compared to 62.3% accuracy for age alone (Table 2).

**Table 2:** Cross-validated logistic regression classification for models trained using cohort status.

	Ball balancing parameters					Age only			
	All	Male	Female	Mean	All		Male	Female	Mean
Accuracy (%)	66.67	72.12	68.93	*70.53* ^a^	*63.47*		66.06	58.57	62.32
Sensitivity (%)	81.82	92.11	78.13	85.12	90.91		95.61	82.03	88.82
Specificity (%)	36.09	27.45	51.22	39.34	13.53		0.00	21.95	10.98
Positive predictive value (%)	69.96	73.94	71.43	72.69	65.67		68.13	62.13	65.13
Negative predictive value (%)	52.17	60.87	60.00	60.43	45.00		0.00	43.90	21.95

^a^Results are shown for a model based on ball balancing inertial measurement unit (IMU) parameters and a model based on age only. The best result per group is italicized. Results for separate gender stratified male and female models are reported as well as models based on All available data.

### Exploratory Results of the FAQ

A small number of ball balancing parameters under both single and dual task conditions were significantly associated with functional impairment (as measured by the FAQ with a threshold of 6), independent of cognitive function (as measured by MMSE total score) and age. These parameters included dual task cost of task performance (percent time in circle), dual task median frequency acceleration and single task radial frequency. A similar analysis controlling for age and RAVLT long recall delay score found that several ball balancing parameters including median frequency acceleration and single task radial frequency were significantly associated with functional impairment.

### RAVLT Exploratory Results

A number of ball balancing parameters under both single and dual task conditions were significantly different on the basis of cognitive status (using RAVLT long recall delay score, with age bucketed thresholds) to define cognitive impairment) and correcting for age.

Pearson correlation coefficient was used to calculate the correlation between the RAVLT (long recall delay score) and each of the calculated ball balancing parameters per task condition. Weak correlations were observed for a number of parameters.

### Classification Using RAVLT Long Delay

A linear logistic regression using RAVLT long recall delay score with age bucket thresholds to denote impairment yielded a mean classification accuracy of 70.43% compared to 57.03% for age alone (Table 3).

Classification results for a model using age alone trained with the RAVLT long delay are also supplied. Results for separate genders stratified as male and female models are reported as well as models based on all available data.

**Table 3:** Classification results for ball balancing classifier model trained using Rey Auditory Verbal Learning Test (RAVLT) long delay score.

	Ball balancing parameters					Age only			
	All	Male	Female	Mean	All		Male	Female	Mean
Accuracy (%)	62.33	76.06	64.80	*70.43* ^a^	54.67		61.21	52.86	57.03
Sensitivity (%)	40.51	30.69	47.87	39.28	74.36		100.00	14.89	57.45
Specificity (%)	30.56	35.94	31.03	33.49	33.33		0.00	83.62	41.81
Positive predictive value (%)	38.73	43.06	36.00	39.53	54.72		61.21	42.42	51.82
Negative predictive value (%)	32.16	24.73	42.35	33.54	54.55		0.00	54.80	54.80

^a^Italics are used to highlight the values most indicative of the true model accuracy.

### Exploratory Results of the MMSE

A number of parameters under both single and dual task conditions were significantly different (using ANOVA and correcting for age) on the basis of cognitive status using MMSE, with a threshold of 28 to classify participants as cognitively impaired or cognitively unimpaired.

Pearson correlation coefficient was used to calculate the correlation between the MMSE (total score) and each of the calculated ball balancing parameters per task condition. Weak correlations were observed for a number of parameters.

### Classification Using the MMSE

A linear logistic regression using the MMSE total score with a threshold of 28 to denote impairment yielded a mean classification accuracy of 72.8% compared to 69.6% for age alone (Table 4).

Classification results for a model using age alone trained with the MMSE are also supplied. Results for separate genders stratified as male and female models are reported as well as models based on all available data.

**Table 4:** Classification results for ball balancing classifier model trained using the Mini Mental State Examination (MMSE) total score.

	Ball balancing parameters										Age only								
	All			M	F		Mean		All				M	F		Mean			
Accuracy (%)		*72.78* ^a^	71.52			72.86		72.19		69.60		67.27			70.48		68.87		
Sensitivity (%)		90.80	94.64			96.64		95.64		99.23		98.21			97.32		97.76		
Specificity (%)		28.95	22.64			14.75		18.70		1.75		1.89			4.92		3.40		
Positive predictive value (%)		74.53	72.11			73.47		72.79		69.81		67.90			71.43		69.66		
Negative predictive value (%)		57.89	66.67			64.29		65.48		50.00		33.33			42.86		38.10		

^a^Italics are used to highlight the values most indicative of the true model accuracy.

## Discussion

We introduce a novel dual task paradigm to evaluate cognitive reserve and prefrontal resource allocation that does not rely on gait and balance metrics and can, thus, be safely completed by older adults and those with falls risk. We found that older adults were able to complete the task regardless of their age or level of cognitive impairment. Even those with MCI and ADRD, as well as those with peripheral neuropathy, osteoarthritis, frailty, and other potential sources of gait and balance problems were able to complete the task reliably and safely.

A sample of 375 participants completed the dual task ball balancing test protocol. Participants ranged in age from 60 to 85 years and exhibited a wide range of cognitive ability. As predicted, participants achieved significantly higher ball balancing test performance under ST conditions (as measured by the percentage of test time, the ball was within the inner circle) compared to DT performance. Thus, along with the higher proportion of perfect tests under ST conditions, the findings confirm that participants were more challenged by the test under DT conditions and that task performance decreased with increasing cognitive impairment. We found that task performance was significantly improved in healthy individuals compared to those with MCI and that performance was worse again in those with ADRD.

A number of significant differences were observed between cognitively intact (unimpaired) and cognitively impaired participants for ball positioning and IMU parameters calculated during a ball balancing test, when correcting statistics for the effect of age, using cohort status, RAVLT long delay score, and MMSE to determine cognitive status.

Significantly decreased performance in the ball balancing test was observed during the DT compared to the ST. Similarly, decreased performance was observed for increasing levels of cognitive impairment. An interpretation of this result is that with increasing impairment, there needs to be greater reliance on cognitive reserve to sustain (or attempt to sustain) cognitive and functional performance. These results are in line with results reported in the literature for other DT paradigms, which suggest that task performance reduced during a DT as compared to an ST and that the reduction in task performance is increased with increased impairment [6,22]. As such, DT performance across different tasks becomes increasingly altered and with that increasingly correlated, while before the high DT cost suggesting impaired reserve (if present at all) might be detectable for some but not all DT conditions. Importantly, a number of ball balancing parameters, measured under both ST and DT conditions were found to be significantly associated with functional impairment (as measured by the FAQ score) independent of MMSE, RAVLT, and age. This suggests that differences observed between MCI to ADRD groups under dual task conditions are consistent with loss of cognitive reserve contributing to progression of clinical manifestation and impact on ADL [4,23]. The ball balancing dual task paradigm may, thus, offer a valuable, objective means to evaluate the risk of ADL impact and enable early detection of MCI-to-dementia transition risk [24].

Moderate classification performance (>70%) was also observed in classifying binary cognitive status using a logistic regression classifier model trained on each of the cognitive function outcome measures. This compared favorably to models based on age alone, which distinguished between impaired and unimpaired groups with ~60% accuracy. A simple linear classifier model (logistic regression) was used to obtain a baseline of classification performance; improved performance may be achieved through the addition of nonlinear interaction terms or the use of higher order classification methods (eg, support vector machines), given the wider data set and potential nonlinear statistical relationships between features. To provide an indication of how well the ball balancing test can distinguish cognitively impaired participants from cognitively intact participants, cross-validation and wrapper-based feature selection was used. This method ensures unbiased estimate of classifier performance on previously unseen participants [25].

Three cognitive function outcome measures were considered in analyzing the use of the ball balancing test in classifying cognitive status. Each outcome measure (MMSE, RAVLT, and cohort status) contains differing and potentially complementary information about cognitive status (as evidenced by the modest mutual correlation observed between each outcome measure). In future work, we will examine the ability of a model based on the weighted combination of the 3 outcomes in longitudinally predicting cognitive impairment on a statistically independent data set. Furthermore, future work may also seek to examine the relationship of the ball balancing test parameters with blood biomarkers [26] and brain structure and pathology [5].

A limitation of this implementation of the ball balancing test is that the virtual ball is not perturbed during the test (other than by the movement of the tablet). This means that placement of the tablet on a flat, stable surface would allow the participant to achieve “perfect” task performance. However, it should be noted that the presence of “perfect score” tests were not found to affect the group-wise findings. An additional limitation is potential usability issues in using this task with an older adult population, particularly those with cognitive fine motor or visual impairments. While the current study involved participants conducting the task under supervised conditions to ensure adequate adherence to the task protocol, there may have been participants in the cognitively impaired groups who struggled to understand the instructions even with the support of the research assistant. Furthermore, impairment to fine motor skills may have prevented some participants from performing to their full capacity. Such usability issues may be exacerbated if the task were to be conducted under unsupervised conditions and would need to be carefully considered in the protocol for future studies.

The ball balancing test is a novel dual task paradigm that may have use in assessment of cognitive reserve and identification of cognitive impairment. Participants with mild or severe cognitive impairment performed less well on the task than healthy participants, particularly when a DT was introduced. A simple cross-validated classifier model used inertial sensor derived parameters obtained during the task to distinguish between cognitively impaired and cognitively intact participants with 70% accuracy. As the ball balancing test can be delivered entirely through a touchscreen tablet device, does not require a controlled environment, and is relatively simple to understand, the task may be suitable for administration by nonexpert users or for unsupervised use in the home environment and could support remote, longitudinal assessment of cognitive function.

## Abbreviations

ADL: activities of daily living
ADRD: Alzheimer disease–related dementia
BDST: backward digit span task
DT: dual task
GAP: Global Alzheimer Platform
IMU: inertial measurement unit
MCI: mild cognitive impairment
MMSE: Mini Mental State Examination
RAVLT: Rey Auditory Verbal Learning Test
ST: single task

## Data Availability

The data sets generated and analyzed during this study are not publicly available due to the terms of the Global Alzheimer Platform (GAP) consortium agreement. However, the data are available from the corresponding author on reasonable request.
